# K_3_VO_2_(V_2_As_2_O_12_)

**DOI:** 10.1107/S1600536809011775

**Published:** 2009-04-02

**Authors:** Safa Ezzine, Mohamed Faouzi Zid, Ahmed Driss

**Affiliations:** a Laboratoire de Matériaux et Cristallochimie, Faculté des Sciences, Université de Tunis-ElManar, 2092 El-Manar, Tunis, Tunisia

## Abstract

A new potassium vanadium arsenate, tripotassium trivanadium bis­(arsenate) hexa­oxide, K_3_VO_2_(V_2_As_2_O_12_), was synthesized by a solid-state reaction at 743 K. The structure is built up from VO_5_ pyramids, VO_4_ tetra­hedra (.*m*. symmetry) and AsO_4_ tetra­hedra linked together by corner-sharing to form a three-dimensional framework. The two crystallographically independent K^+^ cations, one of which has .*m*. symmetry, are located in the inter­connected tunnels running along the **a** and **b** directions.

## Related literature

For preparative details, see: Hajji & Zid (2006 ▶); Ouerfelli *et al.* (2007 ▶); Ben Amor *et al.* (2008 ▶). For structural relationships, see: Haddad & Jouini (1994 ▶); Berrah *et al.* (1999 ▶). For properties of related compounds, see: Lii & Wang (1989 ▶); Lii *et al.* (1990 ▶); Haddad *et al.* (1992 ▶); Aranda *et al.* (1992 ▶); Berrah *et al.* (1999 ▶); Amoros *et al.* (1988 ▶); Leclaire *et al.* (2002 ▶); Daidouh *et al.* (1997 ▶); Nguyen & Sleight (1996 ▶). For bond-valence data, see: Brown & Altermatt (1985 ▶).

## Experimental

### 

#### Crystal data

K_3_V_3_As_2_O_14_
*M* *_r_* = 643.96
Orthorhombic, 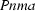

*a* = 7.749 (1) Å
*b* = 16.560 (3) Å
*c* = 10.212 (2) Å
*V* = 1310.4 (4) Å^3^
*Z* = 4
Mo *K*α radiation
μ = 8.13 mm^−1^
*T* = 298 K
0.22 × 0.16 × 0.10 mm

#### Data collection

Enraf–Nonius CAD-4 diffractometer
Absorption correction: ψ scan (North *et al.*, 1968 ▶) *T*_min_ = 0.224, *T*_max_ = 0.444
1950 measured reflections
1451 independent reflections
1210 reflections with *I* > 2s(*I*)
*R*_int_ = 0.025
2 standard reflections frequency: 120 min intensity decay: 1.1%

#### Refinement

*R*[*F* ^2^ > 2σ(*F* ^2^)] = 0.026
*wR*(*F* ^2^) = 0.066
*S* = 1.07
1451 reflections
107 parameters
Δρ_max_ = 0.75 e Å^−3^
Δρ_min_ = −0.55 e Å^−3^

### 

Data collection: *CAD-4 EXPRESS* (Duisenberg, 1992 ▶; Macíček & Yordanov, 1992 ▶); cell refinement: *CAD-4 EXPRESS*; data reduction: *XCAD4* (Harms & Wocadlo, 1995 ▶); program(s) used to solve structure: *SHELXS97* (Sheldrick, 2008 ▶); program(s) used to refine structure: *SHELXL97* (Sheldrick, 2008 ▶); molecular graphics: *DIAMOND* (Brandenburg, 1998 ▶); software used to prepare material for publication: *WinGX* (Farrugia, 1999 ▶).

## Supplementary Material

Crystal structure: contains datablocks I, global. DOI: 10.1107/S1600536809011775/mg2068sup1.cif

Structure factors: contains datablocks I. DOI: 10.1107/S1600536809011775/mg2068Isup2.hkl

Additional supplementary materials:  crystallographic information; 3D view; checkCIF report

## Figures and Tables

**Fig. 1.**
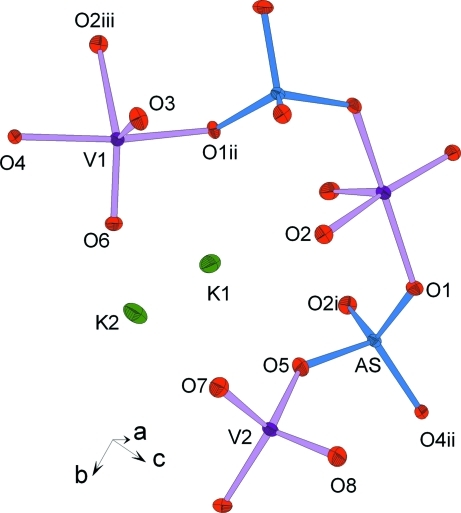
: Unité asymétrique dans K3V3As2O14. Les éllipsoïdes d'agitation thermique ont 50% de probabilité. Codes de symétrie: (i) x - 1/2, y, -z + 3/2; (ii) x, y, z + 1; (iii) -x + 1/2, -y, z - 1/2.

**Fig. 2.**
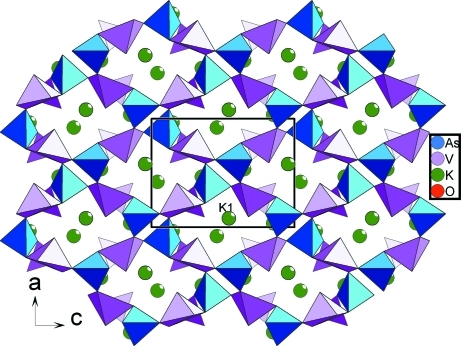
: Projection de la structure de K3V3As2O14 selon b montrant les cavités où logent les ions K1+.

**Fig. 3.**
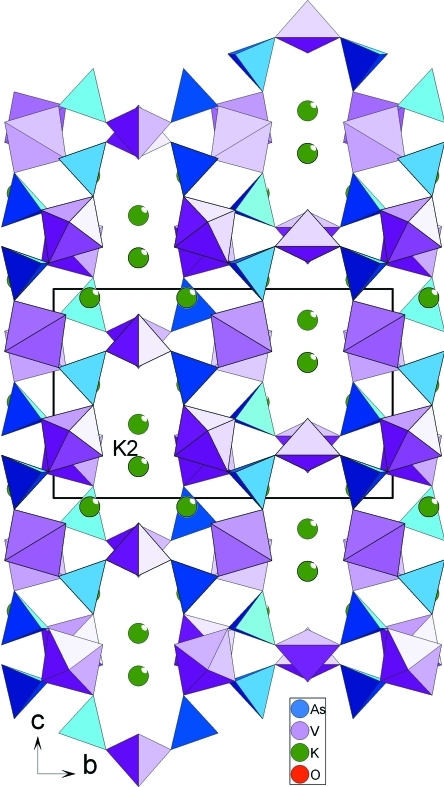
: Projection de la structure de K3V3As2O14 selon a montrant les cavités où logent les ions K2+.

## supplementary crystallographic information

### Comment

La flexibilité des polyèdres VO_n_ et leur association aux tétraèdres XO_4_ (*X* = P ou As) peuvent conduire à des composés possédant des charpentes anioniques ouvertes mixtes unidimensionnelles (Leclaire *et al.*, 2002; Amoros *et al.*, 1988), bidimensionnelles (Lii & Wang, 1989; Lii *et al.*, 1990) et tridimensionnelles (Haddad & Jouini, 1994; Berrah *et al.*, 1999). Ces matériaux à base de métaux monovalents (A = alcalins, Ag) manifestent certaines propriétés physiques intéressantes notamment: de conduction ionique (Daidouh *et al.*, 1997), d'échange d'ions (Aranda *et al.*, 1992) ou parfois catalytique (Nguyen & Sleight, 1996). C'est dans ce cadre que nous avons entrepris l'investigation des systèmes A—V—As—O (A = alcalins, Ag). Un nouveau composé de formulation K_3_VO_2_(V_2_As_2_O_12_) a été synthétisé par réaction à l'état solide à 743 K.

L'unité asymétrique (Fig. 1) dans la structure renferme un tétraèdre VO_4_, deux tétraèdres AsO_4_ et deux pyramides VO_5_ reliés par mise en commun des sommets. L'association des polyèdres AsO_4_ et VO_5_ forme des couches infinies V_2_As_2_O_12_ parallèles au plan (010) (Fig. 2). La jonction entre ces dernières est assurée par partage de sommets entre les tétraèdres V2O_4_ disposés dans l'espace intercouche et les tétraèdres AsO_4_ appartenant aux couches adjacentes. Il en résulte une charpente tridimensionnelle possédant de larges canaux entrecroisés, disposés respectivement selon la direction *a* où se situent les cations K2^+^ (Fig. 3) et selon la direction *b* où logent les cations K1^+^ (Fig. 2). Les atomes d'oxygène non engagés dans les ponts mixtes V—O—As pointent vers les canaux et forment des groupements vanadényl VO_2_.

Le calcul des différentes valences des liaisons utilisant la formule empirique de Brown (Brown & Altermatt, 1985) vérifie bien les valeurs des charges des ions V1(4,99), As(4,95), V2(4,84), K1(1,19) et K2(1,01) dans la phase étudiée.

La comparaison de la structure de K_3_VO_2_(V_2_As_2_O_12_) avec des travaux antérieurs révèle la présence des unités classiques V_2_P_2_O_14_ dans les composés Na_2_V_3_P_2_O_13_ (Haddad et Jouini, 1994) et KV_2_PO_8_ (Berrah *et al.*, 1999) analogues à celles rencontrées dans la phase étudiée. En effet, ces unités se connectent entre elles, d'une part dans KV_2_PO_8_ pour former des rubans qui s'associent au moyen de chaines de pyramides VO_5_ pour constituer une charpente tridimensionnelle de type V_2_PO_8_ et d'autre part dans Na_2_V_3_P_2_O_13_ pour former des couches infinies de formulation V_2_P_2_O_10_. La jonction de ces dernières dans le composé au sodium est réalisée par partage de sommets avec des chaines d'octaèdres VO_6_ et non de tétraèdres VO_4_, comme c'est le cas dans notre composé, pour conduire à une charpente tridimensionnelle. De plus, l'examen des volumes molaires calculés des atomes d'oxygène (volume de la maille/nombre d'atomes d'oxygène par maille) dans ces trois phases conduit respectivement à 19,6 Å^3^ dans Na_2_V_3_P_2_O_13_, 20,6 Å^3^ dans KV_2_PO_8_ et 23,4 Å^3^ dans K_3_VO_2_(V_2_As_2_O_12_) et montre que la charpente anionique dans la phase étudiée est plus ouverte. Cette caractéristique structurale ainsi que la présence de larges cannaux entrecroisés dans notre matériau nous encourage à synthétiser les phases pures de formulation analogues A_3_VO_2_(V_2_As_2_O_12_) (A = Li, Na, K, Ag), procéder aux mesures de la conductivité ionique en fonction de la température par la méthode des impédences complexes et relier ces caractéristiques structurales aux propriétés physico-chimiques (Hajji & Zid, 2006; Ouerfelli *et al.*, 2007; Ben Amor *et al.*, 2008).

### Experimental

Les cristaux relatifs à K_3_VO_2_(V_2_As_2_O_12_) ont été synthétisés à partir du mélange formé de NH_4_H_2_AsO_4_ (préparé au laboratoire, ASTM 01–775), NH_4_VO_3_ (Riedel-De Haën) et KNO_3_ (Fluka). Afin d'éliminer NH_3_, H_2_O et NO_2_, le mélange a été broyé et préchauffé par palier de 150°, suivi de broyages, à l'air jusqu'à 723 K. Il est ensuite porté lentement proche de la fusion jusqu'à 743 K puis abandonné à cette température pendant une semaine pour favoriser la germination des cristaux. Le résidu final a subi d'abord un refroidissement lent (5°/h) jusqu'à 688 K puis un second rapide (50°/h) jusqu'à la température ambiante. Des cristaux jaunâtres, de taille suffisante pour les mesures des intensités, ont été sélectionnés. Une analyse qualitative au M.E.B. de type Philips XL30 confirme la présence des différents éléments chimiques attendus: As, V, et K.

### Crystal data

**Table:**

K_3_V_3_As_2_O_14_	*F*(000) = 1216
*M**_r_* = 643.96	*D*_x_ = 3.264 Mg m^−^^3^
Orthorhombic, *P**n**m**a*	Mo *K*α radiation, λ = 0.71073 Å
Hall symbol: -P 2ac 2n	Cell parameters from 25 reflections
*a* = 7.749 (1) Å	θ = 10–15°
*b* = 16.560 (3) Å	µ = 8.13 mm^−^^1^
*c* = 10.212 (2) Å	*T* = 298 K
*V* = 1310.4 (4) Å^3^	Prism, yellow
*Z* = 4	0.22 × 0.16 × 0.10 mm

### Data collection

**Table:**

Enraf–Nonius CAD-4 diffractometer	1210 reflections with *I* > 2s(*I*)
Radiation source: fine-focus sealed tube	*R*_int_ = 0.025
graphite	θ_max_ = 27.0°, θ_min_ = 2.3°
ω/2θ scans	*h* = −9→1
Absorption correction: ψ scan (North *et al.*, 1968)	*k* = −21→1
*T*_min_ = 0.224, *T*_max_ = 0.444	*l* = −1→13
1950 measured reflections	2 standard reflections every 120 min
1451 independent reflections	intensity decay: 1.1%

### Refinement

**Table:**

Refinement on *F*^2^	Primary atom site location: structure-invariant direct methods
Least-squares matrix: full	Secondary atom site location: difference Fourier map
*R*[*F*^2^ > 2σ(*F*^2^)] = 0.026	*w* = 1/[σ^2^(*F*_o_^2^) + (0.025*P*)^2^ + 1.8132*P*] where *P* = (*F*_o_^2^ + 2*F*_c_^2^)/3
*wR*(*F*^2^) = 0.066	(Δ/σ)_max_ = 0.001
*S* = 1.07	Δρ_max_ = 0.75 e Å^−^^3^
1451 reflections	Δρ_min_ = −0.55 e Å^−^^3^
107 parameters	Extinction correction: *SHELXL97* (Sheldrick, 2008), Fc^*^=kFc[1+0.001xFc^2^λ^3^/sin(2θ)]^-1/4^
0 restraints	Extinction coefficient: 0.0051 (2)

### Special details

**Table:**

Geometry. All e.s.d.'s (except the e.s.d. in the dihedral angle between two l.s. planes) are estimated using the full covariance matrix. The cell e.s.d.'s are taken into account individually in the estimation of e.s.d.'s in distances, angles and torsion angles; correlations between e.s.d.'s in cell parameters are only used when they are defined by crystal symmetry. An approximate (isotropic) treatment of cell e.s.d.'s is used for estimating e.s.d.'s involving l.s. planes.
Refinement. Refinement of *F*^2^ against ALL reflections. The weighted *R*-factor *wR* and goodness of fit *S* are based on *F*^2^, conventional *R*-factors *R* are based on *F*, with *F* set to zero for negative *F*^2^. The threshold expression of *F*^2^ > σ(*F*^2^) is used only for calculating *R*-factors(gt) *etc*. and is not relevant to the choice of reflections for refinement. *R*-factors based on *F*^2^ are statistically about twice as large as those based on *F*, and *R*- factors based on ALL data will be even larger.

### Fractional atomic coordinates and isotropic or equivalent isotropic displacement parameters (Å^2^)

**Table:**

	*x*	*y*	*z*	*U*_iso_*/*U*_eq_	
As	0.10840 (5)	0.07562 (2)	0.90501 (4)	0.00747 (13)	
V1	0.20184 (8)	0.05848 (4)	0.21845 (6)	0.00838 (16)	
V2	0.24092 (12)	0.2500	0.77176 (9)	0.0104 (2)	
K1	0.08189 (12)	0.10726 (6)	0.54286 (9)	0.0174 (2)	
K2	0.46435 (18)	0.2500	0.35139 (14)	0.0203 (3)	
O1	0.2795 (3)	0.01438 (15)	0.8753 (3)	0.0101 (5)	
O2	0.4117 (3)	0.03661 (16)	0.6225 (3)	0.0113 (6)	
O3	0.4050 (4)	0.04567 (16)	0.1786 (3)	0.0152 (6)	
O4	0.1232 (4)	0.11681 (16)	0.0562 (3)	0.0126 (6)	
O5	0.1237 (4)	0.15388 (16)	0.7961 (3)	0.0143 (6)	
O6	0.1942 (4)	0.14453 (16)	0.2994 (3)	0.0127 (6)	
O7	0.3063 (5)	0.2500	0.6193 (4)	0.0164 (9)	
O8	0.4069 (6)	0.2500	0.8710 (4)	0.0195 (9)	

### Atomic displacement parameters (Å^2^)

**Table:**

	*U*^11^	*U*^22^	*U*^33^	*U*^12^	*U*^13^	*U*^23^
As	0.0077 (2)	0.00609 (19)	0.0086 (2)	−0.00023 (14)	−0.00015 (14)	0.00011 (14)
V1	0.0085 (3)	0.0072 (3)	0.0094 (3)	0.0000 (2)	−0.0009 (3)	−0.0002 (2)
V2	0.0136 (5)	0.0064 (4)	0.0111 (5)	0.000	0.0007 (4)	0.000
K1	0.0220 (5)	0.0163 (4)	0.0138 (4)	−0.0007 (4)	0.0004 (4)	−0.0022 (4)
K2	0.0182 (6)	0.0116 (5)	0.0312 (8)	0.000	−0.0029 (6)	0.000
O1	0.0081 (13)	0.0104 (12)	0.0117 (13)	0.0032 (10)	0.0014 (11)	−0.0020 (11)
O2	0.0047 (13)	0.0139 (13)	0.0153 (14)	−0.0009 (11)	0.0009 (10)	0.0008 (11)
O3	0.0112 (14)	0.0168 (14)	0.0178 (14)	0.0026 (11)	0.0032 (11)	0.0047 (12)
O4	0.0202 (14)	0.0094 (12)	0.0083 (12)	0.0051 (12)	−0.0027 (11)	−0.0009 (11)
O5	0.0185 (14)	0.0098 (12)	0.0145 (14)	−0.0007 (11)	−0.0016 (12)	0.0033 (11)
O6	0.0188 (14)	0.0090 (11)	0.0101 (12)	−0.0005 (11)	0.0004 (11)	−0.0009 (10)
O7	0.018 (2)	0.0172 (19)	0.014 (2)	0.000	0.0003 (17)	0.000
O8	0.023 (2)	0.015 (2)	0.020 (2)	0.000	−0.0077 (18)	0.000

### Geometric parameters (Å, °)

**Table:**

As—O2^i^	1.679 (3)	K1—O3^v^	2.834 (3)
As—O4^ii^	1.692 (3)	K1—O1^iii^	2.853 (4)
As—O1	1.696 (3)	K1—O8^i^	2.864 (4)
As—O5	1.712 (3)	K1—O3^vi^	2.889 (5)
V1—O3	1.640 (4)	K1—O1^i^	2.925 (4)
V1—O6	1.648 (3)	K1—O2	2.926 (5)
V1—O1^iii^	2.011 (3)	K1—O7	3.037 (5)
V1—O4	2.012 (3)	K2—O4^vii^	2.696 (4)
V1—O2^iii^	2.053 (4)	K2—O4^viii^	2.696 (4)
V2—O7	1.637 (4)	K2—O6	2.778 (4)
V2—O8	1.637 (5)	K2—O6^iv^	2.778 (4)
V2—O5	1.849 (4)	K2—O6^vii^	2.931 (4)
V2—O5^iv^	1.849 (4)	K2—O6^viii^	2.931 (4)
K1—O6	2.706 (3)	K2—O7	2.997 (4)
K1—O5	2.718 (3)		
			
O2^i^—As—O4^ii^	111.68 (14)	O1^iii^—V1—O4	164.98 (11)
O2^i^—As—O1	116.70 (17)	O3—V1—O2^iii^	101.17 (14)
O4^ii^—As—O1	110.56 (14)	O6—V1—O2^iii^	152.50 (14)
O2^i^—As—O5	104.21 (14)	O1^iii^—V1—O2^iii^	87.18 (14)
O4^ii^—As—O5	106.44 (16)	O4—V1—O2^iii^	81.10 (14)
O1—As—O5	106.39 (15)	O7—V2—O8	110.2 (2)
O3—V1—O6	105.71 (14)	O7—V2—O5	106.24 (12)
O3—V1—O1^iii^	92.92 (13)	O8—V2—O5	107.62 (13)
O6—V1—O1^iii^	97.02 (15)	O7—V2—O5^iv^	106.24 (12)
O3—V1—O4	98.53 (14)	O8—V2—O5^iv^	107.62 (13)
O6—V1—O4	89.24 (15)	O5—V2—O5^iv^	118.8 (2)

Symmetry codes: (i) *x*−1/2, *y*, −*z*+3/2; (ii) *x*, *y*, *z*+1; (iii) −*x*+1/2, −*y*, *z*−1/2; (iv) *x*, −*y*+1/2, *z*; (v) *x*−1/2, *y*, −*z*+1/2; (vi) −*x*+1/2, −*y*, *z*+1/2; (vii) *x*+1/2, −*y*+1/2, −*z*+1/2; (viii) *x*+1/2, *y*, −*z*+1/2.
